# Leveraging Text Mining Approach to Identify What People Want to Know About Mental Disorders From Online Inquiry Platforms

**DOI:** 10.3389/fpubh.2021.759802

**Published:** 2021-10-12

**Authors:** Soowon Park, Yaeji Kim-Knauss, Jin-ah Sim

**Affiliations:** ^1^Department of Education, Kyonggi University, Suwon, South Korea; ^2^Faculty of Humanities, Social Sciences, and Theology, University of Erlangen-Nuremberg, Nuremberg, Germany; ^3^School of AI Convergence, Hallym University, Chuncheon, South Korea

**Keywords:** text mining, mental disorder, online inquiry, latent Dirichlet allocation, topic modeling

## Abstract

Online inquiry platforms, which is where a person can anonymously ask questions, have become an important information source for those who are concerned about social stigma and discrimination that follow mental disorders. Therefore, examining what people inquire about regarding mental disorders would be useful when designing educational programs for communities. The present study aimed to examine the contents of the queries regarding mental disorders that were posted on online inquiry platforms. A total of 4,714 relevant queries from the two major online inquiry platforms were collected. We computed word frequencies, centralities, and latent Dirichlet allocation (LDA) topic modeling. The words like symptom, hospital and treatment ranked as the most frequently used words, and the word my appeared to have the highest centrality. LDA identified four latent topics: (1) the understanding of general symptoms, (2) a disability grading system and welfare entitlement, (3) stressful life events, and (4) social adaptation with mental disorders. People are interested in practical information concerning mental disorders, such as social benefits, social adaptation, more general information about the symptoms and the treatments. Our findings suggest that instructions encompassing different scopes of information are needed when developing educational programs.

## Introduction

Coupled with the rapid expansion of Internet access, individuals have been actively utilizing online inquiry platforms when seeking health information (1). For instance, online inquiry platforms are a unique information source where users could anonymously but still interpersonally exchange sensitive health information. When it comes to mental disorders, using online inquiry platforms have become more salient compared with physical diseases, because the stigmatization of these types of disorders discourage people to ask for a face-to-face consultation (2). Several studies have indeed found this tendency across different cultures (3–5).

Using these online inquiry platforms is especially thriving in the Korean society, which is mostly due to the fact that Koreans are often reluctant to disclose their sensitive health-relevant issues (6). Also, we presumed that one may find online inquiry platforms more favorable due to their features, such as anonymity and accessibility (7). Therefore, exploring these online inquiry platforms where people would frankly open up their concerns and questions would be a great start when examining what people want to know about mental disorders. We expect that refining, classifying, and analyzing the questions made online would contribute to empirical advances in this regard.

### The Biggest Restriction of an Open Communication About Mental Disorders: the Social Stigma

Although the number of people suffering from mental disorders has been growing during the last few decades, the stigmatization of mental disorders is still a global phenomenon that hinders one from receiving proper medical care and treatment (2). Nationwide research involving Korean adults revealed that only about 6% of those who met the criteria for the diagnosis of a mental disorder received medical treatment, and it was discussed that the social stigma played a role in this low rate (8). In fact, a study led by the Seoul National University College of Medicine (2011) (9) has explicitly identified that 18.2% of those who had not received any medical treatment for their mental disorders were concerned about the social stigma following the treatment. Under the circumstances that stigmatization is perceived to prevail, Korean's would rather go online to discuss their health-relevant issues, collect information, and actively interact with other users (6). As such, it is likely that Korean's would prefer using the Internet over a face-to-face support as the main information source when they encounter mental health-relevant issues, so one can remain anonymous and feel less stigmatized (10, 11).

### Online Health Information Seeking Behavior

The Internet may serve a significant supplementary role in the health-relevant decision-making process. People go online before and after seeing their doctor to prepare for, supplement, or validate the consultation (12, 13). In Korea, the Knowledge iN based in Naver (https://kin.naver.com) and the TIP based in Daum-Kakao are the two biggest online inquiry platforms. In fact, an average of 55,000 questions are registered in Knowledge iN on a daily basis (14), and a total of 372 million answers have been accumulated since its first launch in 2002. Their success may be derived from the features of information that integrates experience-based and expertise-based types, even though these types of platforms are generally founded based on a laypersons' subjective experiences regarding the topic (15). In these sites, for example, health professionals are also encouraged to provide answers with the benefit of advertising their workplace for free (16), which attracts people to reveal their sensitive health issues by expecting to have professionals' opinions as well.

Even though there still exist concerns in terms of the quality of information gained online (7), those who worry about the prevailing stigmatization of mental disorders would be inclined to rely on the information provided by these types of online inquiry platforms. A few studies have identified that people sought information online regarding diagnosis, medication, mental health services, and side effects (7, 17, 18). Given the anonymous nature of the Internet, people may find posting sensitive questions online less burdensome than they do it in person. Therefore, examining the contents of these questions would enlighten professionals regarding the needs of their patients and clients, which would then contribute to developing proper intervention plans and educational programs.

### Text Mining as a New Study Tool

By considering this information-seeking tendency as well as the restrictions of open communication, the current study aims to generate a new body of knowledge with the information collected from the online-based inquiry platforms. Using big data could help to find real-world evidence, and therefore to establish a further understanding and development of theories above the classical approaches, such as the survey method (19). Text mining has an incentive as a study methodology in this research (20, 21). Online inquiry platforms guarantee anonymity, and anyone can ask questions without the limitation of time and place, so one can therefore expect that the data gained from these platforms would reflect what people authentically want to know about mental disorders. Employing text mining allows identifying patterns, trends, and relationships that would otherwise remain buried in a large amount of data, which is based on the real-world, yet still unconstructed (22, 23). Previous studies indeed adopted text mining approaches and refined the textual data obtained from online health communities (24), blogs (25), and the published research literature (26) in order to identify patterns of information on diverse topics.

In particular, topic modeling is a useful and popular method for identifying latent topics from a large amount of text data (1). Topic modeling makes use of an algorithm to find topics in a vast unstructured literature group, and is a model for inferring latent topics in a way that groups words with similar meanings via the use of vectors of topic distributions of documents and word distributions of latent topics (27, 28). Topic modeling is recognized as a standard methodology owing to its high degree of performance and convenience. It overcomes many problems facing the analysis of individual word frequency—such as the sparsity problem, synonymy, polysemy, or that of semantic hierarchical structure. For our purposes, we adapted the latent Dirichlet allocation (LDA). LDA assumes that each document can cover a number of topics. In other words, the collected document data is considered a stochastic mixture of these topics, reflecting the nature of the real text. Compared to other topic modeling methods—such as latent semantic analysis (LSA) or probabilistic LSA (pLSA)—results from LDA are easier to interpret (28). In addition, smoothing hyperparameter values into random variables addresses the problem of overfitting (29). Since the current study aimed to examine the latent topics from the inquires of online platforms, we adapted LDA.

### The Current Study Aim

Taking these ideas in conjunction, the current study aimed to investigate queries about mental disorders posted online by using text-mining approaches. As a result, we obtained the questions, which included the words mental disorders, mental disease, mental health, and mental illness, and computed the word frequencies, centralities, and latent Dirichlet allocation (LDA) topic modeling to extract the keywords of the questions and to explore the networks and latent topics thereof.

## Methods

### Data Collection

Queries about mental disorders posted from two representative portal sites in Republic of Korea, which included Naver (https://www.navercorp.com/en/service/featured) and Daum-Kakao (https://www.kakaocorp.com/service/Daum?lang=en) were collected. These two sites provide their own inquiry platforms, which are called Knowledge iN (https://kin.naver.com) and TIP), where people can anonymously ask and answer questions. When it comes to the health/medical category, Naver had the highest market share in 2019 (65.5%), Daum-Kakao ranked third (13.0%), which was following Google (21.1%) (http://www.internettrend.co.kr/trendForward.tsp). We excluded Google, because they provide a different format with the inquiry platform than the other two portal sites do. Using the application programming interfaces (API) provided by Naver and Daum-Kakao, we collected questions about mental disorders in August and November 2019 with the searching keywords of mental disorder, mental disease, mental health, and mental illness. A total of 4,714 queries were collected after deleting the duplicated queries cases.

### Data Analysis

#### Preprocessing

**Figure 1:**
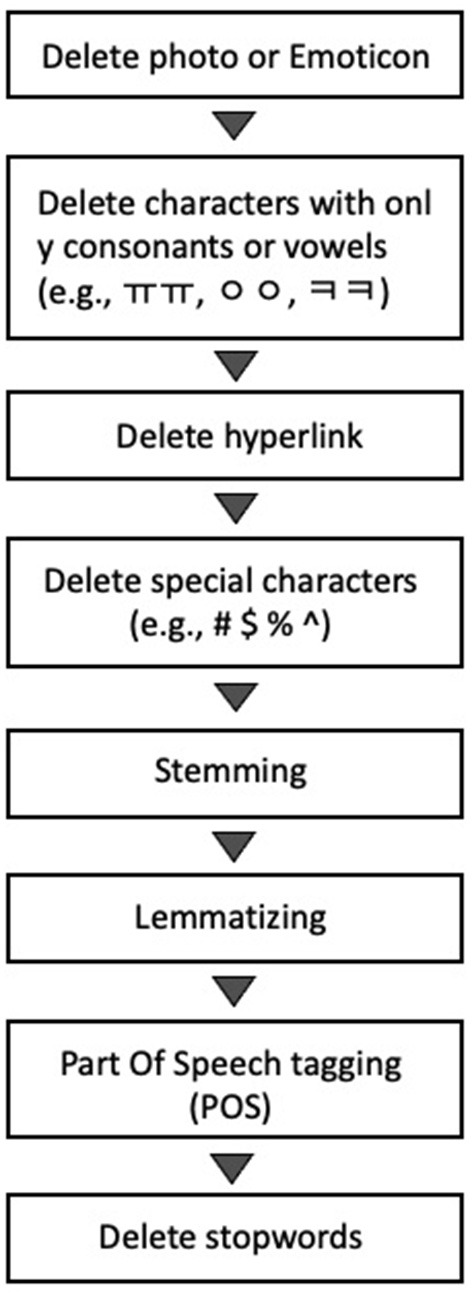
Preprocessing procedure.

For the data preprocessing, photos, emoticons, characters with only consonants or vowels, hyperlinks, and special characters were deleted first. Stemming, lemmatizing, and tagging (part-of-speech tagging, POS) were conducted. We performed a morphological analysis using MeCab from the Python KoNLPy package (30). We first deleted the special characters and formatting tags and then corrected the spellings and the spacing words. After that, we conducted text segmentation, which segments the text into sentences and words, and extracted every noun from the text. The stop words (https://www.ranks.nl/stopwords/korean) were deleted, and the semantically identical words were homogenized using word stemming. All nouns extracted from the text were further analyzed. The preprocessing procedure is presented in Figure 1. After the transformation of the text into mathematical structures, the word frequencies, centralities, and the LDA topic modeling were computed.

#### Centralities

As for the centrality analysis, we calculated four centrality indicators, which included the degree centrality (DC), the betweenness centrality (BC), the closeness centrality (CC), and the eigenvector centrality (EC), to identify relationships between the nodes and to measure the characteristics of the nodes within the network (31). The DC is used to measure the number of links to the other nodes (i.e., nouns), while the EC identifies to what extent a node is directly connected to other more important nodes (32). The CC refers to the inverse of the sum of its distances to all other nodes, and the BC aids to identify the number of times a node bridges the shortest path between two randomly chosen nodes (33).

#### Latent Dirichlet Allocation Topic Modeling

**Figure 2:**
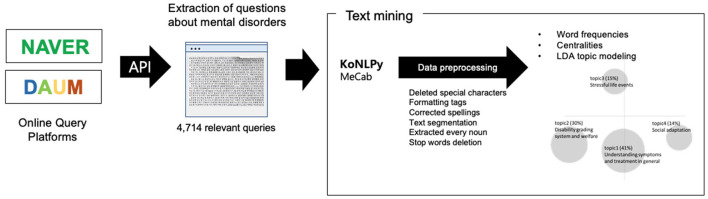
Study procedure.

The LDA is a generative probabilistic framework to model the topical structures of the collected documents, which allows computing the probabilities of each word belonging to the selected topics (34). LDA estimates the posterior distribution of the Bayesian probability model, which determines the percentages of topic compositions in documents and the word compositions of the topics themselves. The meaning of the topic can be inferred from words with high probabilities that constitute the topic in question. The number of topics was determined by referring to the perplexity score, which denotes the difference between the expected value according to the model and the observed value, and the coherence score, which measures the semantic similarities among the words with high probabilities within each topic. The perplexity was calculated from the log-likelihood of a particular word being given as the topic in unseen documents (held-out test set). Lower perplexity and higher coherence imply a better model. Finally, the group discussed and reviewed the plausibility of the suggested model to confirm the final model (Figure 2).

## Results

### Frequencies

The searching keywords, such as mental disorder, mental, and disorder, were ranked high, but we omitted these words from the list presented in Table 1. The words human, symptom, thought, hospital, and treatment were ranked as the five most frequently used words. Regarding the types of mental disorders, depression, anxiety, and schizoid showed high frequencies. Moreover, friend, mother, family, and parents were included within the top forty frequently used words, which represents that the queries concerning one's significant others also appeared frequently.

**Table 1:** Frequencies of the top ranked words.

**Rank**	**Words**	**Frequency**	**Rank**	**Words**	**Frequency**
1	Human	1,235	21	Test	262
2	Symptom	1,091	22	Neurotic	261
3	Thought	971	23	Living	258
4	Hospital	955	24	Doctor	256
5	Treatment	901	25	Women	255
6	Extent	549	26	Condition	249
7	Depression	470	27	Society	248
8	Abnormality	458	28	Diagnosis	236
9	Problem	428	29	Panic	233
10	Cause	422	30	Family	233
11	Friend	402	31	Men	228
12	Anxiety	400	32	School	225
13	Possibility	350	33	Relevance	224
14	Counseling	338	34	Welfare	224
15	Assessment	334	35	Behavior	217
16	Schizoid	333	36	Mind	216
17	Grade	332	37	Time	215
18	Mother	305	38	Ways	215
19	Patients	271	39	Obsession	213
20	Hospitalization	267	40	Parents	213

### Centrality

Four centrality indexes were calculated to examine the network among the extracted words. We found that the word my showed the highest DC (0.78), BC (0.77), CC (0.80), and EC (0.36). Except my and symptom (CC = 0.50), there were not any words that showed coefficients higher than 0.50 across the four centrality indicators. These results indicate that the nodes were hardly connected with each other within the node network.

### Topic Modeling

**Figure 3:**
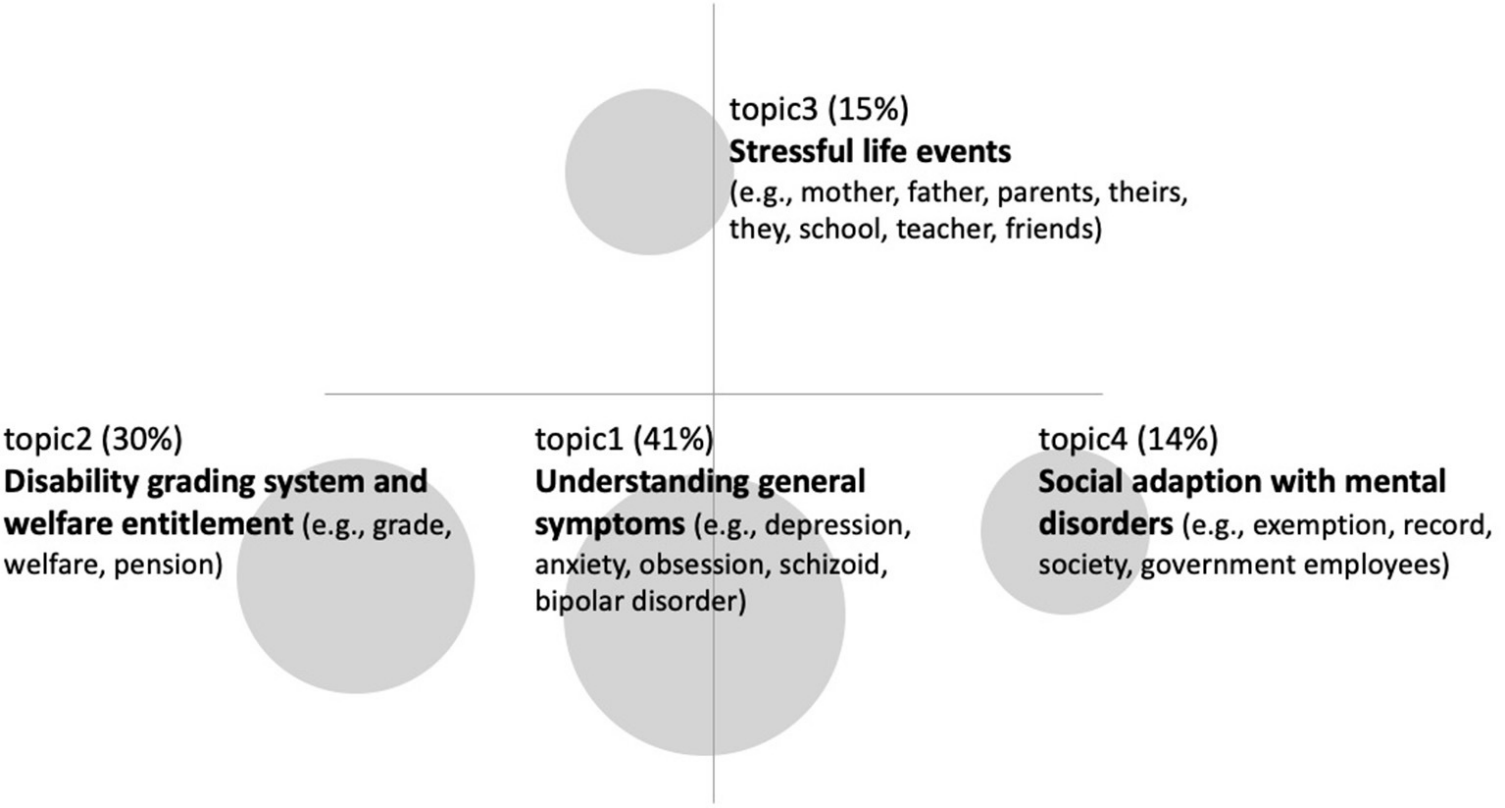
Inter-topic distance map in the LDA model.

We conducted the LDA topic modeling to find the underlying topics by scanning the words and computing their distribution probabilities within the documents (34). Based on the perplexity score, the coherence score, and the group discussion over the plausibility, four topics were selected. Even though the perplexity score was reduced as the number of topics increased, the coherence score reached its peak with the topic number of four. The words and the distribution probabilities in each topic are presented in the Table 2. Based on the contents of the words that belong to the respective topics, we named the four topics as follows. The first topic, which is *understanding general symptoms*, explained 41% of the documents, and it included words that are like the types of mental disorders, which included *depression, anxiety, obsession, schizoid*, and *bipolar disorder, symptoms*, and *treatments*. The second topic, which is *disability grading system and welfare entitlement*, explained 30% of the documents and specifically included the words concerning social benefits, such as *grade, welfare*, and *pension* in conjunction with the contents relevant to the symptoms and the diagnosis. The third topic, which is *stressful life events*, explained 15% of the documents and was comprised of words that included *mother, father, parents, their*s, *they, school, teacher*, and *friends*. The fourth topic, which is *social adaptation with mental disorders*, accounted for 14% of the documents and consisted of the words that included *exemption, record, society, government employees, license*, and *public service worker*. The inter-topic distance map according to the LDA model is presented in Figure 3.

**Table 2:** Four topic models.

**Topic 1 (41%)**		**Topic 2 (30%)**		**Topic 3 (15%)**		**Topic 4 (14%)**	
**Understanding general symptoms**		**Disability grading system and welfare entitlement**		**Stressful life events**		**Social adaption with mental disorder**	
**Words**	**Probability**	**Words**	**Probability**	**Words**	**Probability**	**Words**	**Probability**
Mentality	0.069	Mentality	0.087	Mental disorder	0.026	Mentality	0.050
Disease	0.049	Disability	0.078	Mentality	0.025	Disease	0.026
Mental disorder	0.022	Symptom	0.032	Human	0.021	Hospital	0.024
Disability	0.018	Disease	0.031	Disease	0.013	Treatment	0.020
Thought	0.016	Treatment	0.013	Mother	0.009	Human	0.020
Human	0.011	Neuropathy	0.011	Disability	0.009	Mental disorder	0.012
Treatment	0.011	Counseling	0.009	Symptom	0.008	Disability	0.010
Hospital	0.010	Grade	0.009	Father	0.007	Hospitalization	0.008
Depression	0.010	Hospital	0.008	Problem	0.005	Possibility	0.008
Symptom	0.008	Assessment	0.007	Extent	0.005	Exemption	0.007
Problem	0.008	Human	0.007	Parents	0.005	Months	0.006
Friend	0.007	Diagnosis	0.007	Reason	0.005	Extent	0.006
Anxiety	0.007	Present	0.006	Theirs	0.005	Medicines	0.005
Abnormality	0.006	Psychology	0.006	Stress	0.005	Abnormality	0.005
Extent	0.006	Relevance	0.006	They	0.004	Record	0.005
Cause	0.006	Mental disorder	0.006	Usualness	0.004	Schizoid	0.005
Insurance	0.005	Nervous breakdown	0.006	School	0.004	Health	0.005
Disturbance	0.005	Panic	0.005	Teacher	0.004	Thought	0.005
Patient	0.005	Welfare	0.005	Game	0.004	Society	0.005
Case	0.005	Medical treatment	0.005	Memory	0.004	Psychiatry	0.004
Men	0.005	Test	0.005	Thought	0.004	Government employees	0.004
Causality	0.005	Possibility	0.004	Behavior	0.004	Case	0.004
Women	0.004	Problem	0.004	Hospital	0.004	Full recovery	0.004
Obsession	0.004	Extent	0.004	Cause	0.004	Cause	0.004
Family	0.004	Case	0.004	Friend	0.003	Condition	0.004
Behavior	0.004	Depression	0.004	Abnormality	0.003	Self	0.004
Relation	0.004	Relation	0.004	Time	0.003	Reason	0.004
Schizoid	0.004	Anxiety	0.004	Family	0.003	License	0.004
Stress	0.004	Thought	0.003	Property	0.003	Assessment	0.003
Bipolar disorder	0.004	Living	0.003	Self	0.003	Driving	0.003
Hospitalization	0.003	Cause	0.003	Present	0.003	Test	0.003
Oneself	0.003	Doctor	0.003	Climacterium	0.003	Depression	0.003
Psychiatry	0.003	Intelligence	0.003	Relevance	0.003	Dose	0.003
Auditory hallucination	0.003	Pension	0.003	Depression	0.003	Medical treatment	0.003
Counseling	0.003	Mental retardation	0.003	Addiction	0.002	Employment	0.003
Emotion	0.003	Condition	0.003	Exams	0.002	Military service	0.003
Mind	0.003	Body	0.003	Marriage	0.002	Insurance	0.003
Head	0.003	Society	0.003	Talk	0.002	Public service worker	0.003

## Discussion

### Principal Results

In the present study, we investigated the contents of the queries posted on the two biggest online portal sites in Korea to identify what people authentically want to know about mental disorders. The results revealed that the extracted words were not strongly centered but rather contained varying underlying topics. The results from the topic modeling suggested four topics, which included (1) understanding general symptoms, (2) disability grading system and welfare entitlement, (3) stressful life events, and (4) social adaption with mental disorders. In the following, we will discuss what each topic portrays and how the professionals could employ the current findings for the further development of intervention plans and educational programs.

### Comparison With Prior Work

From the previous review, there were several articles aiming to identify and analyze mental-related symptoms or status (e.g., mental health, anxiety, depression) of online communities using text mining and natural language processing; most of these focused on identification, detection, extraction, and the description of mental-related symptom terms (35–37). While our study focused on general portal online queries, and has tried to observe the patterns of general populations' attitudes and thoughts toward mental-related symptoms or status, those of the articles concerned a variety of both general (e.g., Twitter and patient portals) and disease-specific online communities.

Among the types of mental disorders, the words depression, anxiety, and schizoid showed high frequencies, which were somewhat comparable with the actual prevalence rate of the mental disorder diagnosis as provided by the Ministry of Health and Welfare (38). According to this report, anxiety disorder showed the highest 1-year prevalence rate (5.7%), followed by alcohol use disorder (3.5%), nicotine use disorder (2.5%), depression (1.5%), and schizophrenia spectrum disorder (0.2%). Even though the word depression appeared most frequently in the queries, its actual prevalence rate seems to be relatively low. We presume that these queries may have reflected the depressive mood and the melancholy that one encounters in daily life. On the other hand, the public showed relatively higher interests regarding schizophrenia than it was reported in the prevalence rate. Given that the Korean society was in an uproar over some crimes of schizophrenia during the last few years, this frequency rate might indicate the increased public interest in this topic in general (24).

Besides the types of mental disorders, it should be also noted that words, such as friends, mother, parents, and family were also listed as frequent words, which implies that the queries concern both oneself and one's significant others. As the social relationship functions reciprocally, suffering from mental disorders would be a demanding event for oneself and also for the others who are closely related to the patients. It was in fact reported that the odds of having mental disorders increases by about eight times when their close others suffer from mental disorders (39). Therefore, the environment surrounding a person should be considered when it comes to dealing with mental disorders. Psycho-education to enhance the literacy for mental disorders and the coping strategies against stress would be helpful for both the patients and their close others. It is worth noting that the words mother and parents were ranked higher than wife, husband, daughter, and son. This reflects that younger adults are more likely to post queries and seek help online than older adults do. This may be due to the reason that younger adults are more familiar with using the Internet (40) or that the reported stress level is higher for this age group (41).

The overall coefficients across the four types of centrality were somewhat modest except for one word, which is “my.” The word my had coefficients higher than 0.70 across DC, BC, and CC, which implies that this word was connected with the other nodes directly and indirectly, even though its low EC reveals that there hardly any other influential nodes that existed in this query network. These results of my may reflect that most queries are not general questions, but they are rather personal questions related to oneself, friends, or family. Furthermore, the word symptom showed a relatively higher frequency rate as well as CC compared to the other words, which represents that the information regarding symptoms and treatments might generally be of importance.

The topic modeling showed that the queries consist of four latent topics. According to the LDA results, the queries cover a wide range of topics, which include (1) understanding general symptoms, (2) disability grading system and welfare entitlement, (3) stressful life events, and (4) social adaptation with mental disorders. Even though the earlier works on the online health information-seeking behavior mainly dealt with the contents of general symptom understanding and treatments (7, 17, 18), the topics with a further focus on welfare entitlement, stressful life events, and social adaptation accounted for quite a large proportion of the collected data.

For instance, people sought information to confirm whether one is entitled to receive social benefits due to mental disorders, which is topic 2: disability grading system and welfare entitlement. The words relevant to this topic, such as disability, grade, assessment, test, and pension, seem to have a specific focus on mental disorders as a disability and welfare benefits granted to the registered disabled. Presumably, people seek information regarding the disability grade assessment, which determines the entitlement for the welfare program as well as the extent of the benefits that one could receive. However, this disability grading system has been abolished since July 2019 according to the Fifth Comprehensive Policy Plan for People with Disabilities (2018–2022) (42). Instead, it was replaced by a comprehensive approach in order to ensure the customized welfare services, and this type of detailed information should be further publicized perhaps by employing user-friendly guide materials. In addition to the grading system, the words counseling and treatment are also included in this topic, which is in line with the increased needs of people with mental disorders for welfare services (43).

Regarding topic 3, which is stressful life events, we found that people asked for a general consultation concerning the daily hassles that lead them to feel mentally disturbed. As Yi (6) addressed in the study, this phenomenon may reflect one's expectation that they would be empathized in the first-person in addition to practical and professional advice. Considering that the users of online inquiry platforms were satisfied with the answers that combined both the cognitive and the affective messages (6), practitioners from any platform could optimize the way to deliver the information regarding mental disorders. Moreover, a more accessible channel where one can feel secure to disclose their sensitive health issues should be provided.

On the other hand, topic 4, which is social adaptation with mental disorders, was comprised of the queries that mainly concern the disadvantages and barriers following mental disorders, which could be relevant to the stigmatization thereof (8). For example, the words record, government employees, license, driving, and employment could be understood in terms of the limitation of one's personal and social functioning and in particular the career-wise barriers. Moreover, some society-specific questions were also found. The words exemption, military service, and public service worker may address the possibility of those who are diagnosed with mental disorders to be exempted from the mandatory military service and provide public services instead. Having focused on the topics for young adults, which include job seeking and military service, may be partially due to the average age of the Internet users, but it should be still worthwhile clarifying and providing the information that is particularly relevant to the age- and culture-specific issues.

## Limitations

There are some caveats that should be considered when interpreting our findings. The current study analyzed the texts obtained from online inquiry platforms, which is where people can post questions anonymously. Therefore, any sophisticated distinction by socio-demographic characteristics were not available based on the current dataset. Even though this anonymity was considered to be advantageous when examining what people authentically know about mental disorders, it inevitably hinders planning the interventions to target people with a specific characteristic. Using the dataset that integrates demographical, psychological, or psychiatric information may enrich further discussions in this regard. Furthermore, because the data were collected only on online platforms, the findings may not fully encompass the needs of the Internet illiterate, who might share certain socioeconomic backgrounds or belong to certain age groups. In addition, our analyses would be strengthened by testing the validity of our data, given we had the possibility to examine the same period with similar online platforms.

## Conclusions

This study proposed the use of text-mining approaches to detect queries concerning mental disorders which are posted on online inquiry platforms. The Latent Dirichlet allocation (LDA) topic modeling technique allowed us to successfully extract the keywords of questions relating to mental health problems. Using the real-world data, the findings revealed that the queries, which were posted by those who suffer from mental health problems or those who are close to the patients, covered a wide range of topics such as understanding the general symptoms, a disability grading system, welfare entitlement, stressful life events, and social adaptation with mental disorders. Even though the symptoms and the treatments about mental disorders are still actively discussed, more practical issues that are relevant to one's life, which include social benefits, military service, and employment, also accounted for a large proportion of the collected data. Knowing what people want to know has several theoretical and practical implications for health communication. Future studies will be devoted to investigating more useful mental health issues among the general population. Based on the current findings, one should develop campaigns or educational programs surrounding mental health issues that encompass the diverse scopes of information to effectively help people with mental health issues and to facilitate their social adaptation. Ultimately, the investigation of mental health-related messages from online queries has created many opportunities for improvements to inclusiveness.

## Data Availability Statement

The raw data supporting the conclusions of this article will be made available by the authors, without undue reservation.

## Ethics Statement

Ethical review and approval was not required for the study on human participants in accordance with the local legislation and institutional requirements. Written informed consent for participation was not required for this study in accordance with the national legislation and the institutional requirements.

## Author Contributions

SP conceptualized and designed the study, provided administrative support, assisted in collection and assembly of data, data analysis and result interpretation, and drafted the manuscript. YK-K provided administrative support, assisted in collection and assembly of data, data analysis and result interpretation, and drafted the manuscript. J-aS conceptualized and designed the study, provided administrative support, assisted in the assembly of data, data analysis and result interpretation, and drafted the manuscript. All authors approved the final manuscript as submitted and agree to be accountable for all aspects of the work.

## Funding

This research was supported by Hallym University Research Fund, 2021 (HRF- 202107-001).

## Conflict of Interest

The authors declare that the research was conducted in the absence of any commercial or financial relationships that could be construed as a potential conflict of interest.

## Publisher's Note

All claims expressed in this article are solely those of the authors and do not necessarily represent those of their affiliated organizations, or those of the publisher, the editors and the reviewers. Any product that may be evaluated in this article, or claim that may be made by its manufacturer, is not guaranteed or endorsed by the publisher.

## References

[1] Chu JTW Wang MP Shen C Viswanath K Lam TH Siu S. How, When and why people seek health information online: qualitative study in Hong Kong. Interact J Med Res. (2017) 6:e24. 10.2196/ijmr.7000 29233802 PMC5743920

[2] Hinshaw SP. The stigmatization of mental illness in children and parents: developmental issues, family concerns, and research needs. J Child Psychol Psychiatry. (2005) 46:714–34. 10.1111/j.1469-7610.2005.01456.x 15972067

[3] Park JE Cho SJ Lee JY Sohn JH Seong SJ Suk HW. Impact of stigma on use of mental health services by elderly Koreans. Soc Psychiatry Psychiatr Epidemiol. (2015) 50:757–66. 10.1007/s00127-014-0991-0 25491446

[4] Powell J Clarke A. Internet information-seeking in mental health population survey. Br J Psychiatry. (2006) 189:273–7. 10.1192/bjp.bp.105.017319 16946364

[5] Ybarra ML Eaton WW. Internet-Based mental health interventions. Ment Health Serv Res. (2005) 7:75–87. 10.1007/s11020-005-3779-8 15974154

[6] Yi YJ. Sexual health information-seeking behavior on a social media site : predictors of best answer selection. Online Inf Rev. (2016) 42:880–97. 10.1108/OIR-06-2017-0204

[7] Horgan A Sweeney J. Young students' use of the Internet for mental health. J Psychiatr Ment Health Nurs. (2010) 17:117–23. 10.1111/j.1365-2850.2009.01497.x 20465756

[8] Cho SJ Lee JY Hong JP Lee HB Cho MJ Hahm BJ. Mental health service use in a nationwide sample of Korean adults. Soc Psychiatry Psychiatr Epidemiol. (2009) 44:943–51. 10.1007/s00127-009-0015-7 19294325

[9] Seoul National University College of Medicine. The Epidemiological Survey of Mental Disorders in Korea (2011).

[10] Johnsen J-A Rosenvinge JH Gammon D. Online group interaction and mental health: an analysis of three online discussion forums. Scand J Psychol. (2002) 43:445–9. 10.1111/1467-9450.00313 12500784

[11] Shaw BR Mctavish F Gustafson DH Pingree S. Experiences of women with breast cancer : exchanging social support over the CHESS computer network. J Health Commun. (2000) 5:135–59. 10.1080/108107300406866 11010346

[12] Caiata-zufferey M Abraham A Sommerhalder K Schulz PJ. Online health information seeking in the context of the medical consultation in Switzerland. Qual Health Res. (2010) 20:1050–61. 10.1177/1049732310368404 20442347

[13] Ryu SW Ha YJ. Usage of health information on the internet. Heal Welf Policy Forum. (2004) 11:71–87. 10.1007/s00103-020-03144-5 32367207 PMC8516774

[14] News1.kr. The 13th anniversary of Naver Knowledge iN with accumulative uses of 4.4 million. (2015). Available online at: https://www.news1.kr/articles/?2451685

[15] Song H Omori K Kim J Tenzek KE Hawkins JM Lin WY. Trusting social media as a source of health information: online surveys comparing the United States, Korea, and Hong Kong. J Med Internet Res. (2016) 18:e25. 10.2196/jmir.4193 26976273 PMC4810010

[16] Bae BJ Yi YJ. What answers do questioners want on social Q & A? User preferences of answers about STDs. Internet Res. (2017) 27:1104–21. 10.1108/IntR-08-2016-0245

[17] Aref-adib G Hanlon PO Fullarton K Morant N Sommerlad A Johnson S. A qualitative study of online mental health information seeking behaviour by those with psychosis. BMC Psychiatry. (2016) 16:232. 10.1186/s12888-016-0952-0 27400874 PMC4940927

[18] Kalckreuth S Trefflich F Rummel-kluge C. Mental health related internet use among psychiatric patients : a cross-sectional analysis. BMC Psychiatry. (2014) 14:368. 10.1186/s12888-014-0368-7 25599722 PMC4299476

[19] Chen EE Wojcik SP. A practical guide to big data research in psychology. Psychol Methods. (2016) 21:458–74. 10.1037/met0000111 27918178

[20] Delespierre T Denormandie P Bar-Hen A Josseran L. Empirical advances with text mining of electronic health records. BMC Med Inform Decis Mak. (2017) 17:127. 10.1186/s12911-017-0519-0 28830417 PMC5568397

[21] Kao A Poteet SR. Natural Language Processing and Text Mining. New York, NY: Springer; (2007).

[22] Gupta V Lehal GS. A survey of text mining techniques and applications. J Emerg Technol Web Intell. (2009) 1:60–76. 10.4304/jetwi.1.1.60-76

[23] Harlow LL Oswald FL. Big data in psychology to the special issue. Psychol Methods. (2016) 21:447–57. 10.1037/met0000120 27918177 PMC5221569

[24] Park A Conway M Chen A. Examining thematic similarity, difference, and membership in three online mental health communities from reddit: a text mining and visualization approach. Comput Human Behav. (2018) 78:98–112. 10.1016/j.chb.2017.09.001 29456286 PMC5810583

[25] Yarkoni T. Personality in 100,000 words: a large-scale analysis of personality and word use among bloggers. J Res Pers. (2010) 44:363–73. 10.1016/j.jrp.2010.04.001 20563301 PMC2885844

[26] Singh N Hu C Roehl WS. Text mining a decade of progress in hospitality human resource management research: identifying emerging thematic development. Int J Hosp Manag. (2007) 26:131–47. 10.1016/j.ijhm.2005.10.002

[27] Steyvers M Griffiths T. Probabilistic topic models. In: Handbook of Latent Semantic Analysis. (2007). p. 424–40.

[28] Blei DM. Probabilistic topic models. Communications. (2012) 55:77–84. 10.1145/2133806.2133826

[29] Griffiths TL Steyvers M. Finding scientific topics. Proc Natl acad Sci USA. (2004) 101:5228–5235. 10.1073/pnas.0307752101 14872004 PMC387300

[30] Park L. KoNLPy Documentation (2019).

[31] Freeman LC. Centrality in social networks conceptual clarification. Soc Netws. (1978) 1:215–39. 10.1016/0378-8733(78)90021-7

[32] Behera RK Rath SK Misra S Damasevicius R Maskeliunas R. Distributed centrality analysis of social network data using mapreduce. Algorithms. (2019) 12:161. 10.3390/a12080161

[33] Tanaka K Takahashi M Tsuda K. Comparison of centrality indexes in network Japanese text analysis. Int J e-Educ e-Bus e-Manag e-Learn. (2013) 3:37–42. 10.7763/IJEEEE.2013.V3.189

[34] Blei DM Ng AY Jordan MI. Latent Dirichlet allocation. J Mach Learn Res. (2003) 3:993–1022. 10.5555/944919.944937

[35] Calvo RA Milne DN Hussain MS Christensen H. Natural language processing in mental health applications using non-clinical texts. Nat Lang Eng. (2017) 23:1–37 10.1017/S1351324916000383

[36] Dodd M Janson S Facione N Faucett J Froelicher ES Humphreys J. Advancing the science of symptom management. J Adv Nurs. (2001) 33:668–76. 10.1046/j.1365-2648.2001.01697.x 11298204

[37] Feldhege J Moessner M Bauer S. Who says what? Content and participation characteristics in an online depression community. J Affect Disord. (2020) 263:521–7. 10.1016/j.jad.2019.11.007 31780138

[38] Hong JP Lee DW Ham BJ Lee SH Sung SJ Yoon T. The Survey of Mental Disorders in Korea. Sejong (2017).

[39] Bambauer KZ Zhang B Maciejewski PK Sahay N Pirl WF Block SD. Mutuality and specificity of mental disorders in advanced cancer patients and caregivers. Soc Psychiatry Psychiatr Epidemiol. (2006) 41:819–24. 10.1007/s00127-006-0103-x 16865636 PMC2504328

[40] Korean Educational Statistics Service. Adolescents' Internet and Smartphone Usage. (2019). Available online at: https://kess.kedi.re.kr/post/6684806?itemCode=03&menuId=m_02_03_03

[41] Choi I Choi J Choi E Lee S Kim N Lee S. About H: Korea Happiness Report. Paju: Book21 (2019).

[42] Kim SH. The comprehensive policy plan for people with disabilities: progress and challenges. Heal Welf Policy Forum. (2018) 285:62–71.

[43] Yoon SY. Welfare service use in people with disabilities: Current state and policy implications. Heal Welf Policy Forum. (2018) 263:50–60.

